# High Doses of Melatonin Induce Seizure-like Cortical Activity: Electrophysiological and Behavioral Evidence

**DOI:** 10.1021/acsomega.6c00727

**Published:** 2026-05-05

**Authors:** Lorena Almeida, Axell Lins, Luciana Eiró-Quirino, Luana V. de Souza, Maria K. O. Hamoy, Priscille F. P. Hartcoff, Marcelo V. dos S. Brito, Gabriela B. Barbosa, Thaysa de S. Reis, Moisés Hamoy

**Affiliations:** Laboratory of Pharmacology and Toxicology of Natural Products, 37871 Federal University of Pará (UFPA), Belém, Pará 66075-110, Brazil

## Abstract

Melatonin is widely used as a neurohormone and dietary supplement for sleep regulation and is generally regarded as safe in therapeutic doses. However, its neuropharmacological effects at supraphysiological concentrations have remained insufficiently characterized. In the present study, we investigated the behavioral and electrophysiological consequences of high-dose melatonin administration and examined the ability of classical anticonvulsant drugs to modulate its excitatory effects. Adult male Wistar rats received melatonin (100 mg/kg, ip), and behavioral responses were systematically recorded. Cortical activity was assessed by electrocorticography (ECoG) with spectral analysis of delta to γ frequency bands. Additional experimental groups received PTE, PBT, or DZP during the excitatory phase to evaluate pharmacological control of seizure-like activity. Melatonin induced a reproducible biphasic profile, characterized by an initial sedative and myorelaxant phase, followed by marked cortical hyperexcitability accompanied by tremors and clonic seizures. ECoG recordings revealed an increase in signal amplitude and power spectral density during the excitatory phase, particularly within β and γ oscillations, consistent with ictal-like activity. The administration of anticonvulsant agents significantly attenuated these electrophysiological alterations and reduced the behavioral manifestations of hyperexcitability. These results demonstrate that melatonin, at high doses, can shift from an inhibitory to a proexcitatory neuropharmacological profile, likely involving underlying mechanisms of disruption of central nervous system homeostasis. The study highlights a paradoxical action of melatonin on cortical excitability, raising concerns about the potential for intoxication in susceptible patients, such as children who are at risk.

## Graphical abstract

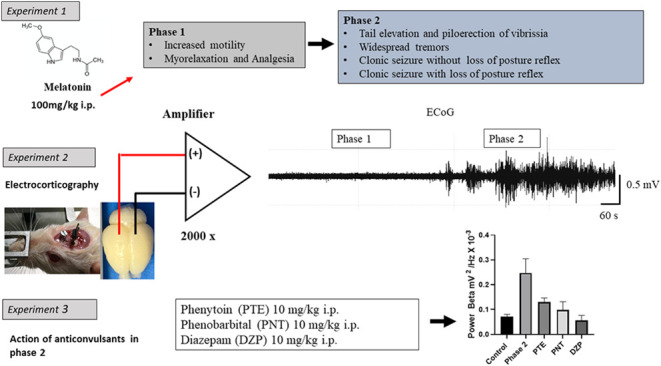

## 1 Introduction

Melatonin is a hormone produced by the body and secreted mostly by the pineal gland in a circadian cycle. This hormone acts on the activity-rest and sleep-wake cycles and is responsible for inducing sleep in all stages, including rapid eye movement (REM) sleep.^1–3^ Its synthesis is inhibited in the presence of light, and with technological advancement, artificial lights compromise the production of melatonin and lead individuals to develop various sleep-related disorders.^4–8^

Thus, exogenous melatonin supplementation has become popular as an alternative for the treatment of sleep disorders.^9–11^ The lack of obvious adverse effects at low doses in the short and long-term, the good tolerance of the organism unchallenged, and the low potential for dependence, make the marketing and use of melatonin highly indiscriminate.^12–15^ According to Vural et al.,^12^ the dose of melatonin varies from 0.1 mg to 50 mg/kg when administered orally, revealing an increase in melatonin levels. Because it is a fat-soluble molecule that easily crosses the blood–brain barrier, melatonin modulates brain activity. Its benefits can be cited in several articles.^16–21^

This study presents a unique neuropharmacological perspective on melatonin, demonstrating, for the first time, that supraphysiological doses can alter its classic inhibitory profile, resulting in seizure-like activity. Unlike previous research focused mainly on the antioxidant, chronobiotic, or anticonvulsant properties of melatonin, our approach combines detailed behavioral assessment with electrocorticographic (ECoG) monitoring to capture real-time neurophysiological alterations across multiple frequency bands. The integration of pharmacological modulation with PTE, PBT, and DZP further reinforces the evidence that the excitatory effects of melatonin involve dysregulation among neurotransmitters that promote homeostasis in the central nervous system.

The importance of the present study is underscored by the widespread over-the-counter availability of melatonin, which may promote the misconception that it is an entirely harmless substance. The absence of mandatory medical supervision facilitates excessive or inappropriate use, including toxicological dosing, a concern that is particularly relevant to pediatric populations. Melatonin is often marketed in palatable formulations such as syrups, chewable tablets, or gummies, increasing the risk of accidental overconsumption by children. Notably, experimental and clinical evidence indicate that high doses of melatonin may lower the epileptic seizure threshold, especially in individuals with an underlying neurological vulnerability. In children predisposed to epilepsy, such dose-dependent effects may enhance the cortical hyperexcitability and trigger seizures. By demonstrating that supraphysiological melatonin induces significant electrophysiological alterations and seizure-like activity, this study challenges the perception of melatonin as an innocuous compound and highlights the need for greater regulatory oversight, clearer dosing guidelines, and increased public awareness regarding its potential neurological risks.

## 2 Experiments

### 2.1 Materials and Methods

#### 2.1.1 Animals

A total of 42 adult albino male Wistar rats, weighing between 200 and 290g, from the central vivarium of Federal University of Pará (UFPA), Belém—PA, Brazil, and maintained in the Laboratory of Toxicology and Pharmacology of Natural Products in the same institution, with feed and water *ad libitum*, were used, with an ambient temperature of 23–25 °C and a 12-h light–dark cycle (lights go out at 6:00 PM and come back on at 6:00 AM). All experimental procedures were performed in accordance with the principles of laboratory animal care (NIH Publication No. 86-23, revised in 1985). All experimental procedures involving animals were approved by the Institutional Animal Care and Use Committee of Federal University of Pará under protocol number No. 6525040822 (ID 002003) and were conducted in accordance with the NIH Guide for the Care and Use of Laboratory.

#### 2.1.2 Chemicals

The anesthetic ketamine 100 mg/mL (dose 100 mg/kg i.p.) was acquired from the König laboratory (Santana de Parnaíba, SP, Brazil) and the xylazine 20 mg/mL (dose 10 mg/kg i.p.) from the Vallée laboratory (Montes Claros, MG, Brazil), while the local anesthetic lidocaine 2% 20 mg/mL (dose 0,3 mL local) was obtained from Hipolabor (Sabará, MG, Brazil). Melatonin (Marketed formulation) was acquired from Cosmed Industria de Cosméticos e Medicamentos S.A., Rua VPR-3, Anápolis, Goiás, Brazil.

##### 2.1.2.1 Experiment 1—Behavioral Characterization

The behavior (*n* = 9) was observed after the administration of 100 mg/kg i.p. of melatonin; in this case, linear regression data were used for the appearance of clonic seizures with loss of posture reflex. At the dose used, the animals presented two phases. Phase 1 was characterized by decreased mobility, myorelaxation, and anesthesia (evaluated through anesthesia in the dorsal region of the right scapular region). Then, phase 2 represented by a period of excitability, with tail flagging and vibrissae, generalized tremor, clonic seizure without loss of posture reflex, and clonic seizure with loss of posture reflex. During the behavioral test, the animals were kept in standard white cages (60 × 50 × 40 cm) for a period of 30 min. All behavioral experiments were conducted in the morning between 7:30 and 11:00 AM. Light/dark cycle: light period 6:00–18:00 h and dark period 18:00–6:00 h. Melatonin was administered between 8:30 and 10:00 in the morning to avoid interfering with the circadian rhythm and the release of cortisol, which in rodents occurs in the afternoon.

##### 2.1.2.2 Experiment 2—Experimental Groups

Three doses of intraperitoneal melatonin were tested: 50, 75, and 100 mg/kg. All caused drowsiness and seizures; however, the 100 mg/kg dose was necessary because it provided clearer phases in the analyses, allowing for better behavioral observation, and these phases began less than 15 min after administration. Furthermore, the 100 mg/kg i.p. dose is 10 times higher than the anticonvulsant dose of 10 mg/kg.^44^

All records were made in the morning, from 7:30 am to 11:00 am. The following groups were formed: (a) Control group (without sleep deprivation): The animals received application of 0.9% saline solution, in the volume of 1 mL i.p., then the records were made in the animals awake and moving (*n* = 9); (b) Sleep control group: they received application of 0.9% saline solution, in the volume of 1 mL i.p., and the records were made with the animals asleep (*n* = 9); and (c) Melatonin Group: the animals were treated with 100 mg/kg i.p., then electrocorticographic recordings were made. Each recording lasted 30 min (*n* = 9).

The animals endured 24 h of sleep deprivation in cages with platforms 3 cm in diameter and 6 cm high, containing a 2 cm deep layer of water in cages measuring 60 × 50 × 40 cm.

##### 2.1.2.3 Experiment 3—Evaluation of Anticonvulsant Drugs in Phase 2

The animals initially received 100 mg/kg i.p. melatonin and were monitored electrocorticographically for possible convulsive graphoelements. Next, 10 mg/kg i.p. of anticonvulsants: PTE, Phenobarbital, and Diazepam were applied to evaluate the efficacy in controlling seizures. The groups were distributed as follows: (a) Group treated with phenytoin (PTE) (*n* = 9); (b) Phenobarbital (PBT) group (*n* = 9), and (c) Diazepam (DZP) group (*n* = 9). The records lasted 15 min.

#### 2.1.3 Electrode Placement Surgery

The animals were anesthetized by intraperitoneal injection of ketamine hydrochloride (100 mg/kg) and xylazine hydrochloride (10 mg/kg). After the abolition of the corneal reflex, the animals were placed in a stereotaxic device. After surgical procedures to expose the skull, two bilateral holes were drilled into the rat’s skull with a dental drill. 925 silver electrodes (tip exposure 1.0 mm in diameter) were placed in the dura mater, above the occipital cortex at the coordinates of the bregma −5.20 mm and ± 1.0 mm laterally.^22^ A screw was fixed in the frontal region of the skull, and the electrodes were fixed with dental acrylic cement. The left electrode was used as the reference electrode, and the right-side electrode was used as the recording electrode in a bipolar system.

#### 2.1.4 Electroencephalographic Recordings and Analysis of the Records

After surgery, the animals were kept in individual cages for a period of 5 days. Subsequently, the electrodes were connected to a digital data acquisition system composed of a high-impedance amplifier (Grass Technologies, P511), an oscilloscope (Protek, 6510), and a data acquisition and digitization board (National Instruments, Austin, TX). The data were shown continuously at 1 kHz at a low pass of 3 kHz and a high pass of 0.3 Hz.

Offline analysis was performed using a tool built using the Python programming language (version 2.7). The “Numpy” and “Scipy” libraries were used for mathematical processing, and the “matplotlib” library was used to obtain graphs and plots. A graphical interface was developed by using the PyQt4 library. The spectrograms were calculated by using the Hamming window with 256 points (256/1000 s). For the power spectral density (PSD), each frame was generated with an overlap of 128 points per window. For each frame, the PSD was calculated by using the Welch mean periodogram method. Frequency histograms were obtained by calculating the PSD of the signal using the Hamming window with 256 nonoverlapping points, resulting in a resolution of 1 Hz per bin. Each wave displayed in PSD is an average of a set of experiments. PSDs were calculated in each group, and the averages are shown by individual boxes (Figure 1D). The analyses were performed at a frequency of up to 40 Hz and divided into bands according to Jalilifar et al.^23^ into delta (1–4 Hz), theta (4–8 Hz), α (8–12 Hz), β (12–28), and γ (28–40 Hz) for interpretation of brain dynamics after treatment.^24^

**Figure 1:**
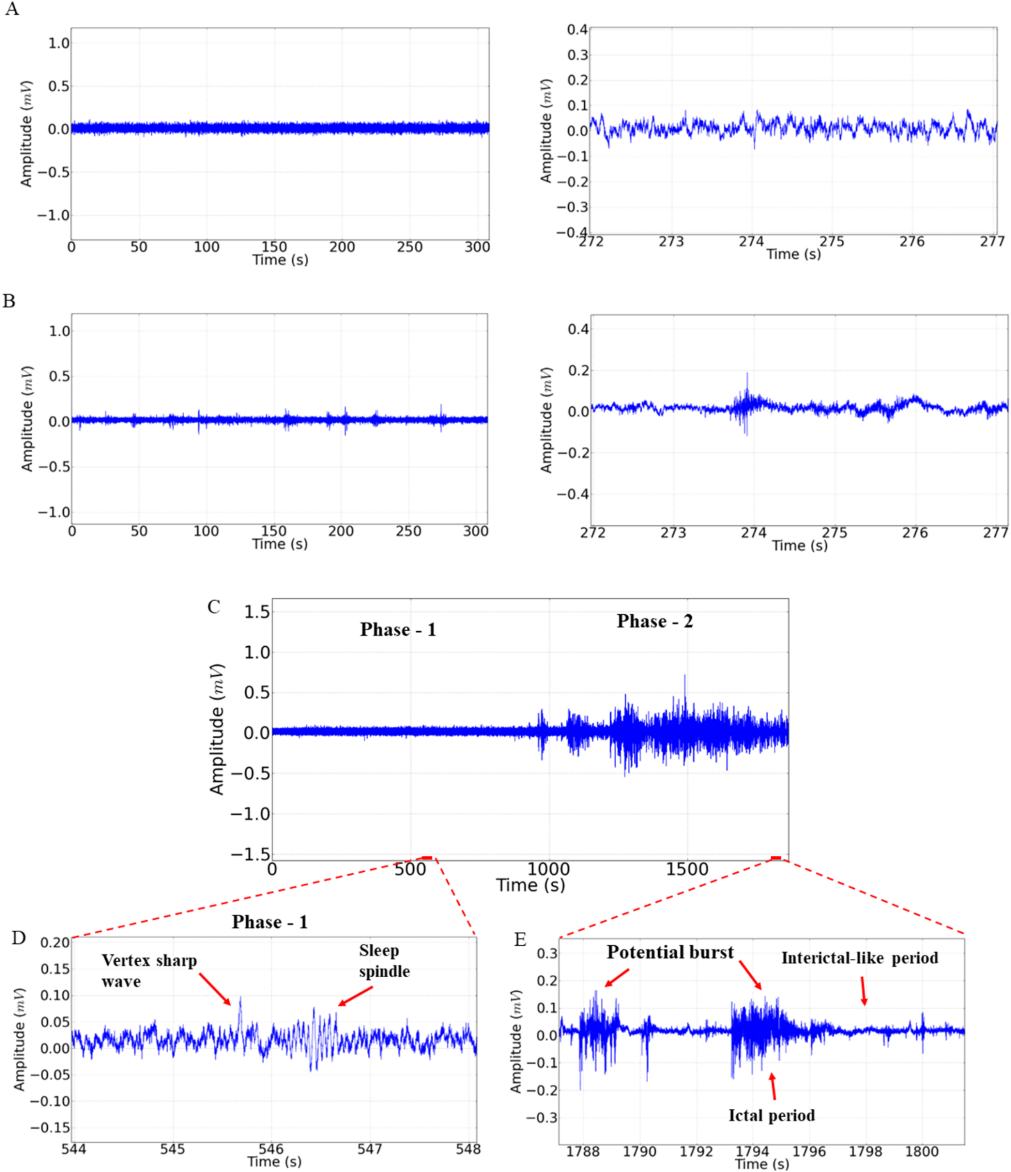
Electrocorticographic tracings (ECoG) from Wistar rat to control group (left), amplification in 5 s in the period 272–277s from the record (right) (A); electrocorticographic tracing of the sleeping animal (left), enlarged record in the period 272–277s of the record (right) (B); demonstrative record of the group submitted to treatment with 100 mg/kg i.p. of melatonin (C), phase 1 amplification, with indication of the acute vertex wave and sleep spindle (D) and phase 2 amplification with indication of graphoelements (red arrow) with potential cluster characteristic, with representation of the ictal period and postictal-like period (E).

#### 2.1.5 Statistical Analyses

The normality and homogeneity of the variances were verified by the Kolmogorov–Smirnov and Levene tests, respectively. Analyses were performed using one-way analysis of variance (ANOVA) and Tukey’s counter-test. The GraphPad Prism 9 software was used for the statistical tests. The results were expressed as mean and standard deviation, and the significance levels were *p < 0.05, **p < 0.01, and ***p < 0.001.

## 3 Results

The behavioral observations obtained after the administration of melatonin at a dose of 100 mg/kg i.p. were characterized by two phases. The first phase of sleepiness and analgesia, followed by phase 2 of CNS excitability (Table 1). In phase 1, it is possible to perceive two distinct behaviors: decreased mobility and myorelaxation with analgesia. The decrease in mobility began after about 10 min, with a latency of 546.4 ± 64.44 s. Myorelaxation with analgesia, in turn, began at around 15 min, with a latency of 822.0 ± 120.8 s (Table 1).

**Table 1:** Latency in Seconds for the Onset of Behaviors after Melatonin Administration, Phase 1—Sleep and Analgesia, and Phase 2—Excitability (*n* = 9) Can Be Observed

phase	behavior	latency (s)
phase 1	increased motility	546.4 ± 64.44
	myorelaxation and Analgesia	822.0 ± 120.8
phase 2	tail elevation and piloerection of vibrissae	1033 ± 60.95
	widespread tremors	1276 ± 97.19
	clonic seizure without loss of posture reflex	1457.0 ± 89.38
	clonic seizure with loss of posture reflex	1627 ± 44.80

The second phase begins about 18 min after the application of melatonin and is characterized by excitability and the appearance of a flagged tail and bristling of vibrissae. The seizure observed in the animals progressed to generalized tremors, followed by clonic seizure without loss of posture reflex, but which rapidly worsens to clonic seizure with loss of posture reflex, with a latency of 1627 ± 44.80 s (Table 1).

### 3.1 ECoG Confirmed Two Phases of Brain Activity after Melatonin Treatment

The animals in the control group walked normally, presenting ECoG characteristics with low tracing amplitude (Figure 1A left). Graphoelements with low amplitude and predominant characteristics of oscillations in β (12–28 Hz) were observed (Figure 1A left). The group sleeping in deep sleep presented irregularities in the tracings (Figure 1B left), and these irregularities can be evidenced from the amplification of the tracing, which demonstrates periods of frequency acceleration and presentations of small sleep spindles (Figure 1B left).

For the animals treated with melatonin, two phases were observed. Phase 1 occurred soon after the application of melatonin with low amplitude maintenance (Figure 1C); however, when enlarged, acute waves of vertex and sleep spindle can be observed as graphoelements (Figure 1D). During Phase 2, much larger amplitude traces can be noted (Figure 1C), which are characterized by potential salvoes. During the amplification of the tracing, ictal periods (IPs) and postictal-like periods (ILPs) can be identified, and the presence of these graphoelements is cyclic, as shown in Figure 1E.

The variation in linear energy power at frequencies up to 40 Hz, between the records in phases 1 and 2, was an increase in power in phase 2 (1.584 ± 0.256 mV^2^/Hz × 10^–3^), being higher than in the other groups. The control group (0.4975 ± 0.09097 mV^2^/Hz × 10^–3^) was like the sleep groups (0.3988 ± 0.07113 mV^2^/Hz × 10^–3^) (*p* = 0.4675) and phase 1 (0.4404 ± 0.0487 mV^2^/Hz × 10^–3^) (*p* = 0.830). The sleep group was like phase 1 (*p* = 0.9254) (Figure 2A).

**Figure 2:**
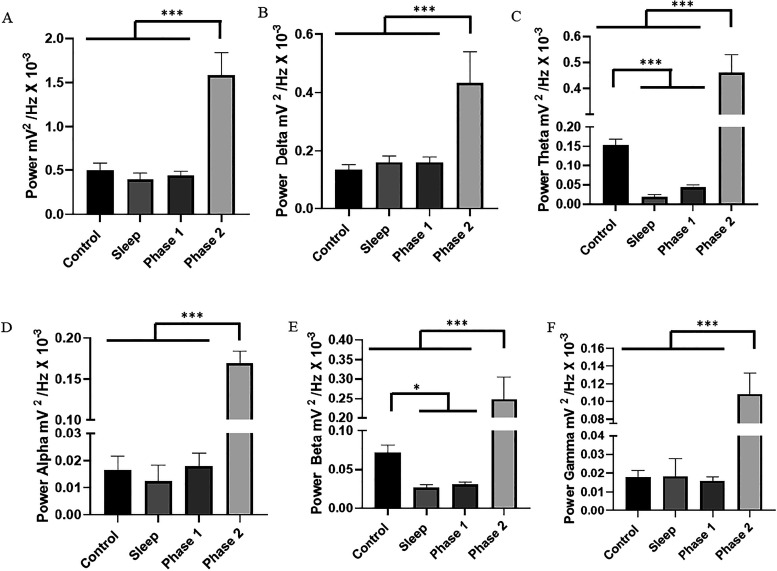
Quantitative distribution of linear frequency of brain waves at frequencies up to 40 Hz to evaluate power between control, sleep, phase 1 and phase 2 (A); oscillations in delta (1–4 Hz) (B); oscillations in theta (4–8 Hz) (C); oscillations in α (8–12 Hz) (D); oscillations in β (12–28 Hz) (E) and oscillations in γ (28–40 Hz) (F). The test used was unidirectional ANOVA. Data are expressed as mean ± SD (*n* = 9 animals per group; *p < 0.05, **p < 0.01, and ***p < 0.001).

For the cerebral oscillations in delta (1–4 Hz), phase 2 (0.433 ± 0.1069 mV^2^/Hz × 10^–3^) presented a higher mean power than the other groups. The control group (0.133 ± 0.0183 mV^2^/Hz × 10^–3^) was like the sleep groups (0.1595 ± 0.02345 mV^2^/Hz × 10^3^) (*p* = 0.7506) and phase 1 (0.160 ± 0.01936 mV^2^/Hz × 10^–3^) (*p* = 0.9738). The sleep group was similar to phase 1 (*p* = 0.9999) (Figure 2B).

The oscillations in theta (4–8 Hz) mean power for the control group (0.1539 ± 0.01481 mV^2^/Hz × 10^–3^) were higher than the sleep group (0.01856 ± 0.00644 mV^2^/Hz × 10^–3^) and phase 1 (0.04398 ± 0.006 mV^2^/Hz × 10^–3^). The phase 1 group was like the sleep group (*p* = 0.4439). The phase 2 group (0.461 ± 0.0693 mV^2^/Hz × 10^–3^) was superior to the other groups (Figure 2C).

In the oscillations between 8 and 12 Hz, in α, phase 2 (0.1696 ± 0.04167 mV^2^/Hz × 10^–3^) was superior to the other groups. The control groups (0.0165 ± 0.00495 mV^2^/Hz × 10^–3^), sleep group (0.0124 ± 0.00590 mV^2^/Hz × 10^–3^), and phase 1 (0.0179 ± 0.004691 mV^2^/Hz × 10^–3^) were like each other (*p* = 0.5529) (Figure 2D).

For the oscillations in β (12–28 Hz), the mean power for the control group (0.0718 ± 0.00911 mV^2^/Hz × 10^–3^) was higher than the sleep group (0.02653 ± 0.00356 mV^2^/Hz × 10^–3^) and phase 1 (0.0306 ± 0.00275 mV^2^/Hz × 10^–3^). Phase 1 had a mean power like that of the sleep group (*p* = 0.990). The phase 2 group (0.247 ± 0.05705 mV^2^/Hz × 10^–3^) was superior to the other groups (Figure 2E).

For the cerebral oscillations in γ (28–40 Hz), phase 2 (0.1086 ± 0.0233 mV^2^/Hz × 10^–3^) presented a higher mean power than the other groups. The control group (0.01784 ± 0.00376 mV^2^/Hz × 10^–3^) was like the sleep groups (0.01822 ± 0.00958 mV^2^/Hz × 10^–3^) and phase 1 (0.01579 ± 0.00958 mV^2^/Hz × 10^–3^) (*p* = 0.9864). The sleep group was similar to phase 1 (*p* = 0.9776) (Figure 2F).

### 3.2 Analysis of Brain Oscillations during the Ictal Period and Postictal Period Indicates a Variation in the Power of Brain Activity

During the analysis of the records in phase 2, periods of rapid power change were detected, characterizing the ictal period (IP) and the postictal-like period (ILP) (Figure 1E). The IP group (2.90 ± 0.247 mV^2^/Hz × 10^–3^) showed superior power compared to the control group, phase 2, and ILP. The control group (0.497 ± 0.0909 mV^2^/Hz × 10^–3^) was like the ILP group (0.294 ± 0.294 mV^2^/Hz × 10^–3^) (*p* = 0.1118) (Figure 3A).

**Figure 3:**
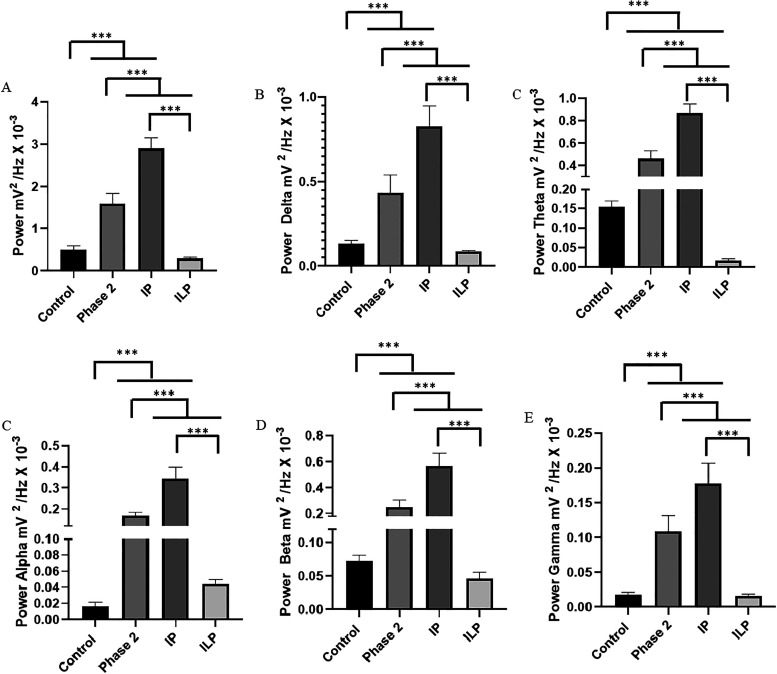
Quantitative distribution of linear brain wave frequencies at frequencies up to 40 Hz to compare the power between the ictal period and the postictal-like period in phase 2 (A), delta oscillations (1–4 Hz) (B); oscillations in theta (4–8 Hz) (C); oscillations in α (8–12 Hz) (D); oscillations in β (12–28 Hz) (E) and oscillations in (28–40 Hz) (F). The test used was unidirectional ANOVA. Data are expressed as mean ± SD (*n* = 9 animals per group; *p < 0.05, **p < 0.01, and ***p < 0.001).

There was an increase in delta wave frequencies for the treated animals, presenting IP (0.826 ± 0.1204 mV^2^/Hz × 10^–3^), higher than the other groups. The control group (0.133 ± 0.01831 mV^2^/Hz × 10^–3^) was like the ILP (0.085 ± 0.0062 mV^2^/Hz × 10^–3^) (*p* = 0.6033). Phase 2 had a higher mean than the control group and the ILP period (Figure 3B).

The oscillations in theta had a higher average power for the IP (0.866 ± 0.0822 mV^2^/Hz × 10^–3^) in relation to the other groups. The ILP period (0.01741 ± 0.0043 mV^2^/Hz × 10^–3^) presented the lowest mean power in relation to the other groups (Figure 3C).

For the oscillations in α, the ictal period showed a higher mean power (0.342 ± 0.056 mV^2^/Hz × 10^–3^) than the other groups. The control group was like the ILP group (0.0441 ± 0.0054 mV^2^/Hz × 10^–3^) (*p* = 0.2089). The group in phase 2 was longer than the ILP period (Figure 3D).

For the oscillations in β, the mean power during the IP (0.5686 ± 0.00973 mV^2^/Hz × 10^–3^) was higher than that of the other groups. In the period of energy decrease (ILP) (0.0461 ± 0.00982 mV^2^/Hz × 10^–3^), it was like the control group (*p* = 07718) and lower than phase 2 (Figure 3E).

The average γ oscillations during the high energy period of the records (IP) (0.1775 ± 0.0291 mV^2^/Hz × 10^–3^) were higher than those of the other groups. The control group was like the period of lowest energy level in the register (ILP) (0.0159 ± 0.00284 mV^2^/Hz × 10^–3^) (Figure 3F).

### 3.3 Evaluation of Anticonvulsant Medications

The evaluation of seizure control in phase 2 was performed through electrocorticographic monitoring after administration of anticonvulsants at the time of the seizure that occurred after melatonin administration. The groups were treated with the following anticonvulsants: PTE (10 mg/kg i.p.), PBT (10 mg/kg i.p.), and DZP (10 mg/kg i.p.). The recording patterns obtained with the use of anticonvulsants are shown in Figure 4A–C.

**Figure 4:**
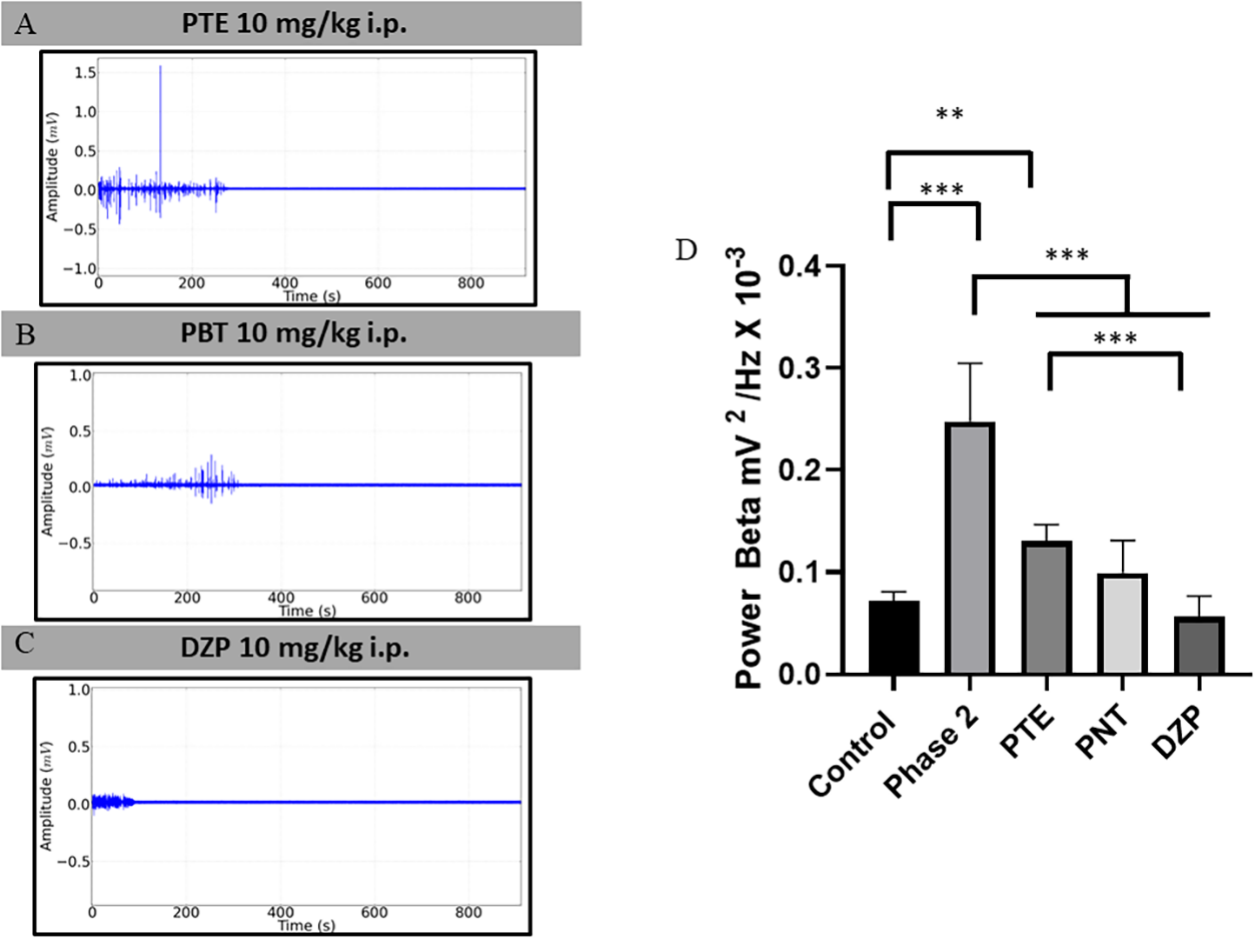
Electrocorticographic recordings (ECoG) obtained in Phase 2 in β brain oscillations after anticonvulsant application. Demonstration of the registration pattern found after application of PTE 10 mg/kg i.p. (A); PBT at 10 mg/kg i.p. (B); DZP at 10 mg/kg i.p. (C) and mean potency values showing the action of anticonvulsants during phase 2. Tests used: ANOVA and Tukey’s test (*n* = 9). *p < 0.05; **p < 0.01, and ***p < 0.001.

Among the brain oscillations, the one that is most related to seizures is the β frequencies, which showed an increase during phase 2. Thus, the analysis of oscillations in β was evaluated after treatment with anticonvulsants. For the control group, the mean power was 0.0718 ± 0.00911 mV^2^/Hz × 10^–3^ and increased during Phase 2 to 0.2478 ± 0.057 mV^2^/Hz × 10^–3^. After the use of anticonvulsants, the records of the animals showed a reduction in amplitude. The PTE-treated group (0.1308 ± 0.0158 mV^2^/Hz × 10^–3^) was like the PBT group (0.0994 ± 0.0318 mV^2^/Hz × 10^–3^) (*p* = 0.2363). The group treated with PBT was like the control group (*p* = 0.3605). The group treated with DZP (0.0568 ± 0.01968 mV^2^/Hz × 10^–3^) was similar to the control (0.8511) and PBT (*p* = 0.05010) (Figure 4D) groups.

## 4 Discussion

The results found from the experiments carried out demonstrate that the group treated with melatonin generated two responses in the behavioral evaluation, later ratified and observed in the analysis of brain wave frequencies. In phase 1, it was possible to observe the presence of graphoelements such as acute vertex waves and sleep spindles, common in some stages of sleep, and which may be associated with transitions in the sleep state or with initial effects of melatonin that affect brain activity in a way similar to sleep onset or an altered state of relaxation.

The altered biophysical profile of cells may constitute a possible mechanism underlying the proposed beneficial effects of melatonin in brain-related disorders, such as epilepsy. Specifically, melatonin reduced firing frequency and threshold potential remained unchanged.^25,26^ However, some studies have shown inconclusive evidence regarding the anticonvulsant effect of melatonin.^27–31^ These initial data corroborate what has been described in the literature. Nakaoka et al.^32^ state in their study that melatonin induces sleep in a way similar to physiological sleep, without added effects, making it beneficial when compared to other hypnotic classes. The study by Salanitro et al.^33^ in people of different ages who have various sleep-related disorders points out that melatonin was significant in improving sleep latency and total sleep time. In phase 1, sleep induction and myorelaxation, like that observed, occurred, but with the evolution of behavior, seizure excitability was observed.

From the amplification of ECoG, it is possible to observe that during phase 2, there is an ictal period and a postictal-like period, suggesting that after the initial effect of melatonin to induce sleep, there is a disturbance in the brain’s electrical activity of excitability considered excessive or abnormal, like epileptic seizures and/or recovery after a seizure.

It is possible that exogenous melatonin in high doses results in a more active postsleep state than observed under normal conditions or that a “rebound effect” occurs, where the brain, after being inhibited by a high dose of melatonin, experiences a compensatory increase in its activity after reduced melatonin action. This is in line with several articles that deal with the anticonvulsant action of melatonin.^34–39^

Conversely, studies indicate that melatonin has low toxicity and is considered safe compared with other drugs commonly used to induce sleep. In this study, the author states that, at doses above 300 mg, melatonin does not present adverse reactions in adults, apart from an increase in drowsiness.^1^ They also add that melatonin is highly lipid-soluble, confirming its benefits to the CNS, as well as its safety and tolerability, even at high doses.^40^

Another study explains several positive effects on the body caused by melatonin;^41^ however, its use without proper medical guidance, without proven need or without sanitary guarantee can lead to undesirable side effects, even if mild and transient.^42^ These effects may include headache, irritability, abdominal discomfort, fatigue, dizziness, skin rashes, psychotic effects, and difficulty concentrating, among others.^41^

The use of melatonin as a supplement or medication is to improve sleep quality, especially in children who produce less melatonin, but it does not have a direct effect on inducing sleep. There are several studies that indicate melatonin as an anticonvulsant in rodents using a dose of 10 mg/kg.^28,35,43,44^ In our work, it was observed that 10 times the anticonvulsant dose induced clonic seizures in the animals.

There is also the possibility that the high concentration of melatonin causes nonspecific effects on multiple neurotransmitter systems, affecting not only sleep-related receptors but also other circuits that modulate various responses in the body.^40^

A review showed that 200 cases of adverse events were reported in France, of which 43% were neurological disorders (seizure, syncope, and headache), 24% were psychiatric disorders (anxiety and depression), 19% were skin disorders (skin rashes and maculopapular rashes), and 19% were digestive problems (constipation, nausea, and acute pancreatitis).^42^

The results obtained in the experiments indicate that, although melatonin is already widely used as a safe and low-toxicity substance, in high doses, it can cause brain changes similar to seizures. This suggests that, in high or accidental doses, potentially dangerous effects on the central nervous system may occur, which have not been reported to date. However, the administration time, between 8:30 and 10:00 in the morning, should be taken into consideration to avoid the cortisol peak, since studies have observed variable patterns of sensitivity to melatonin depending on the circadian clock, allowing for modifications in responses in channels that may be related to excitability.^45–48^

Although there are articles that contradict the results found and declare the safe use of the substance, it is essential to consider the milder adverse effects reported by other authors. The discrepancy between studies suggests that the effects of the substance may vary depending on the dose and individual factors of the users. Therefore, as it is an over-the-counter product often used as a supplement, caution is recommended in patients who may increase the dose ingested on their own and control access, especially for children.

## 5 Conclusions

It is also important to highlight the need for further research to better understand the mechanisms of action and potential health risks associated with the use of melatonin, especially at high doses. The results of this experiment challenge the common perception of safety associated with the recurrent and indiscriminate use of melatonin, emphasizing the importance of rigorous dose control. Thus, the development of clear clinical guidelines could contribute to ensuring the safe and effective use of this substance as a sleep inducer or an adjunct to other therapies.

Although this study provides strong evidence that high doses of melatonin can induce seizure-like cortical activity, some limitations should be considered. The use of a single high-dose regimen prevents the establishment of a complete dose–response relationship, recording only one brain location (motor cortex). Furthermore, the study was conducted exclusively in male Wistar rats, and potential sex-dependent differences still need to be investigated. Future studies should explore pharmacodynamics at the receptor level, the effects of long-term exposure, and molecular biomarkers of excitability to better elucidate the pathways underlying melatonin’s actions in inducing central nervous system excitability. Despite these limitations, the current results highlight an important and previously under-recognized aspect of melatonin pharmacology, emphasizing the need for greater control over self-medication and increased caution regarding melatonin access, particularly by children and domestic animals.

## Data Availability

All data generated or analyzed during this study are available in the public repository at the following link: https://drive.google.com/file/d/158Twymg6aQp32BggkrjXWryrpPPIYBEt/view?usp=sharing. The data sets support the findings of this study and include the original electrophysiological and behavioral data.
